# The impact of preoperative sarcopenia on postoperative ileus following colorectal cancer surgery

**DOI:** 10.1007/s10151-023-02812-3

**Published:** 2023-05-15

**Authors:** L. Traeger, S. Bedrikovetski, TM. Nguyen, Y. X. Kwan, M. Lewis, J. W. Moore, T. Sammour

**Affiliations:** 1 Colorectal Unit, Department of Surgery, Royal Adelaide Hospital, Port Road, Adelaide, SA 5000 Australia; 2 Adelaide Medical School, Faculty of Health and Medical Sciences, University of Adelaide, Adelaide, SA Australia

**Keywords:** Sarcopenia, Postoperative ileus, GI-2, Colorectal surgery

## Abstract

**Purpose:**

Sarcopenia is associated with poor short- and long-term patient outcomes following colorectal surgery. Despite postoperative ileus (POI) being a major complication following colorectal surgery, the predictive value of sarcopenia for POI is unclear. We assessed the association between sarcopenia and POI in patients with colorectal cancer.

**Methods:**

Elective colorectal cancer surgery patients were retrospectively included (2018–2022). The cross-sectional psoas area was calculated using preoperative staging imaging at the level of the 3rd lumbar vertebrae. Sarcopenia was determined using gender-specific cut-offs. The primary outcome POI was defined as not achieving GI-2 by day 4. Demographics, operative characteristics, and complications were compared via univariate and multivariate analyses.

**Results:**

Of 297 patients, 67 (22.6%) were sarcopenic. Patients with sarcopenia were older (median 74 (IQR 67–82) vs. 69 (58–76) years, *p* < 0.001) and had lower body mass index (median 24.4 (IQR 22.2–28.6) vs. 28.8 (24.9–31.9) kg/m^2^, *p* < 0.001). POI was significantly more prevalent in patients with sarcopenia (41.8% vs. 26.5%, *p* = 0.016). Overall rate of complications (85.1% vs. 68.3%, *p* = 0.007), Calvien-Dindo grade > 3 (13.4% vs. 10.0%, *p* = 0.026) and length of stay were increased in patients with sarcopenia (median 7 (IQR 5–12) vs. 6 (4–8) days, *p* = 0.013). Anastomotic leak rate was higher in patients with sarcopenia although the difference was not statistically significant (7.5% vs. 2.6%, *p* = 0.064). Multivariate analysis demonstrated sarcopenia (OR 2.0, 95% CI 1.1–3.8), male sex (OR 1.9, 95% CI 1.0–3.5), postoperative hypokalemia (OR 3.2, 95% CI 1.6–6.5) and increased opioid use (OR 2.4, 95% CI 1.3–4.3) were predictive of POI.

**Conclusion:**

Sarcopenia demonstrates an association with POI. Future research towards truly identifying the predictive value of sarcopenia for postoperative complications could improve informed consent and operative planning for surgical patients.

## Introduction

Sarcopenia has become increasingly recognized as a significant contributor to poor short- and long-term patient outcomes following colorectal surgery [1–4]. Sarcopenia is the development of frailty associated with advanced aging and results in a reduction of muscle mass and/or function [5, 6]. Patients with colorectal malignancy have sarcopenia compounded as a result of the pronounced effects of malignancy on metabolism, and neoadjuvant therapy resulting in reduced appetite, vomiting and pain [5, 6]. In a recent meta-analysis, 37% of patients with colorectal cancer had sarcopenia, and this was associated with an increase in severe postoperative complications and mortality [3]. As preoperative staging computed tomography (CT) is routinely performed, the cross-sectional area of the psoas can be easily determined to quantify lean muscle mass, offering an opportunity to diagnose and to potentially intervene in high-risk patients with sarcopenia [7, 8].

Postoperative ileus (POI) occurs in up to 25% of cases following colorectal resection [9–11]. With paralysis of the gastrointestinal (GI) tract, patients experience abdominal distention, diet intolerance, constipation, nausea, and vomiting, increasing risk of complications such as pneumonia and delayed wound healing [12, 13]. Furthermore, POI increases the risk of renal and hepatic failure, prolongs hospital stay, and increases 30-day readmission [12, 13]. As a result of this POI poses a significant financial burden to patients and healthcare services, increasing total hospital costs by 26% in Australia and 50–100% worldwide [14–17].

Despite the high prevalence of preoperative sarcopenia and POI as a complication in colorectal surgical patients, there is limited literature establishing a connection between them. POI is well known to be associated with advanced age and significant comorbidities [18]. Similarly, sarcopenia has a strong link to geriatric syndromes and constipation [19]. It has also been speculated that diet imbalances related to sarcopenia impair macrophage response to peritoneal irritation, resulting in POI [20]. Trejo-Avila et al. identified 23 papers investigating postoperative colorectal complications in patients with sarcopenia; however, only four articles reported POI as a complication [21–24]. These studies did not define POI, had low sample sizes and reported a disproportionately low incidence of POI in a colorectal cohort where POI is expectedly common. Of these studies, one demonstrated a higher incidence of POI in the sarcopenic group; however, the assessment was limited by sample size (25% vs. 17.4%, *n* = 47) [23]. Noting the lack of literature on the predictive value of sarcopenia, Sasaki et al. investigated the correlation between skeletal muscle index (SMI) and POI [25]. Using bioelectrical impedance analysis to quantify SMI, they found that low SMI was associated with an increased likelihood of developing POI (HR 10.8 (95% CI 1.25–93.20), *p* = 0.031), independent of age and sex. There remains a significant gap in the literature investigating the incidence of POI and the return of gastrointestinal function using validated composite measures and strict clinical definitions in patients with sarcopenia following colorectal surgery.

This study aims to assess the role of sarcopenia as a predictor for POI following colorectal surgery.

## Methods

This study was approved by the Central Adelaide Local Health Network (CALHN) Human Research Ethics Committee with a waiver of consent for retrospective studies and is reported using the Strengthening The Reporting of Observational studies in Epidemiology (STROBE) guidelines [26].

### Patient selection

This study was performed at the Colorectal Unit of the Royal Adelaide Hospital (RAH), a tertiary referral centre in South Australia, Australia. Patients who underwent elective colorectal cancer surgery between January 2018 and June 2022 were identified through the prospective colorectal cancer database of patients discussed at the weekly RAH Colorectal Cancer multidisciplinary meeting. All patients at the RAH are placed on an enhanced recovery pathway (ERP) postoperatively. The ERP protocol can be found at www.tinyurl.com/raheras.

### Inclusion and exclusion criteria

Consecutive elective colorectal patients over 18 years old who underwent major bowel surgery, consisting of large bowel resection for colorectal malignancy, were included. Patients were excluded if they had undergone emergency surgery, small bowel resection, defunctioning ostomy without bowel resection, colonic stenting, transanal endoscopic microsurgery (TEMS), care was delivered at a different facility or had non-operative management. Patients were also excluded if they had insufficient data or had not undergone staging CT accessible by using our local Picture Archiving and Communication System (PACS) or InteleViewer™ Australia.

### Sarcopenia calculation

**Fig. 1:**
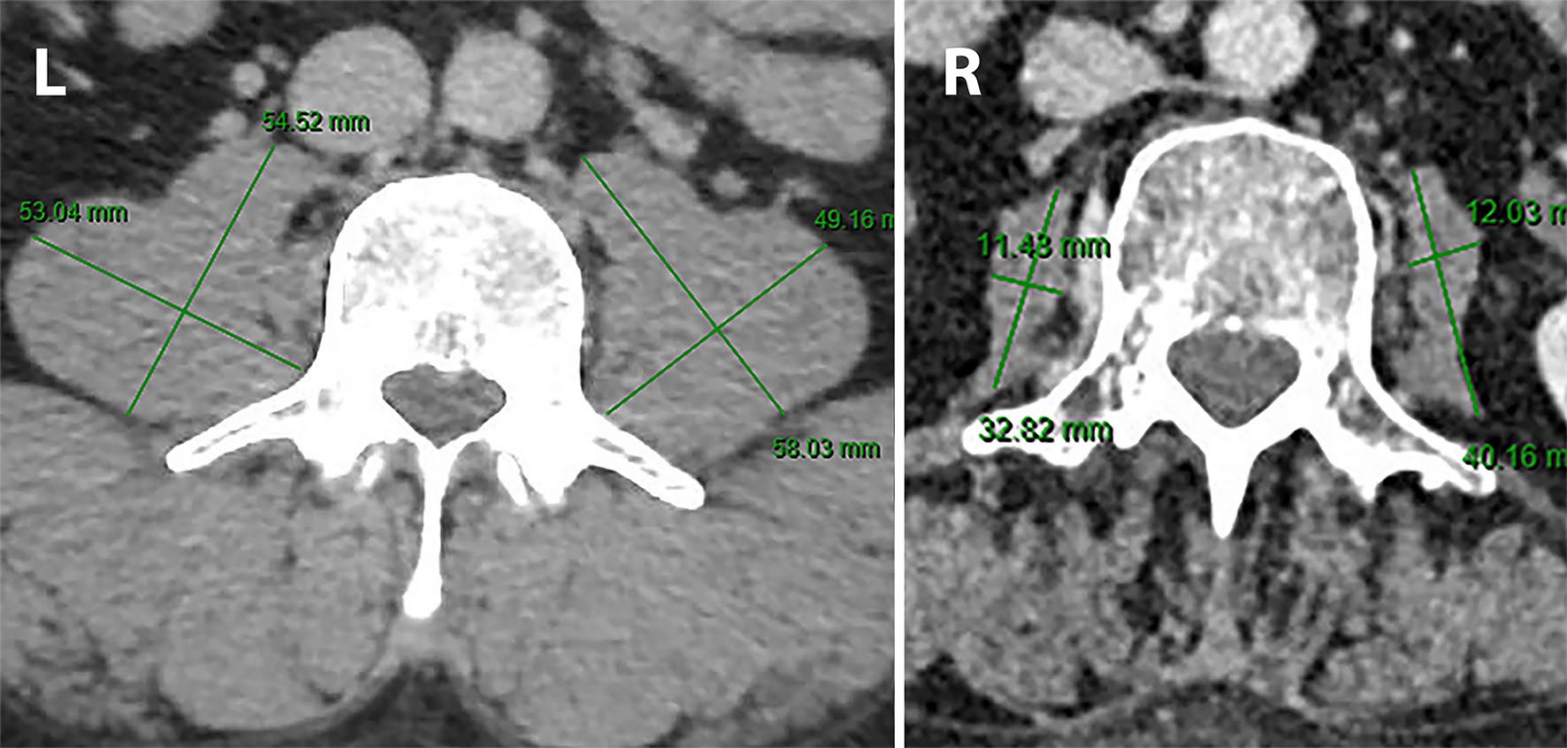
Example assessment of a patient without sarcopenia (left) and with sarcopenia (right) via psoas muscle measurement

Lean muscle mass was calculated using the protocol defined by Jones et al. using preoperative staging CT scans [8]. Staging CT scans were retrieved from the time of diagnosis or post neoadjuvant therapy prior to operation, whichever came later. Total psoas area (TPA) was calculated by multiplying the longest anterior–posterior (AP) and transverse muscle diameters bilaterally (Fig. 1). Measurements were taken at the level of the third lumbar vertebrae using PACS or InteleViewer™ Australia. This method of AP and transverse muscle diameter calculation has been proven comparable to computer-calculated TPA (*r*^2^ 0.944 (95% CI 0.86–0.97), *p* = 0.001) [8]. TPA was normalized for the patient’s height squared (TPAmm^2^/m^2^) to calculate the total psoas area index (TPAI). Sarcopenia was defined using previously validated gender-specific cut-off points: < 385 mm^2^/m^2^ in women and < 545 mm^2^/m^2^ in men [27]. Two investigators (LT and SB) independently calculated lean muscle mass for all patients to identify sarcopenia. Conflicting results were resolved by consensus.

### Data collection

Medical records were reviewed retrospectively. Known risk factors for the development of POI were collected [9–11, 14, 28]. Baseline demographics included age, gender, height, weight, body mass index (BMI), American Society of Anesthesiologists (ASA) score, American Joint Committee on Cancer (AJCC) stage and preoperative neoadjuvant treatment. Components of the modified frailty index (MFI)-5 (i.e. patient functional status, and medical history of congestive cardiac failure, chronic obstructive pulmonary disease, diabetes mellitus and hypertension requiring medication) were collated as the MFI-5 has been associated with frailty and poor surgical outcomes following colorectal surgery [4]. Other nutritional data, including total protein and albumin, were also collected. Operative data included surgical approach (open/laparoscopic), conversion rates, procedure type, stoma formation, duration of surgery, and perioperative intravenous fluid administration. Postoperative data included requirements in morphine equivalents (intraoperative, postoperative recovery and day 1–4) calculated using Opioid Calculator v2.9.1 (Faculty of Pain Medicine, Australian and New Zealand College of Anaesthetists, Australia).

### Outcomes

The primary outcome was POI, defined by not achieving GI-2 by postoperative day 4, a threshold suggested by Vather et al. [29]. GI-2 is a validated outcome measure comprising time to first stool and tolerance of solid diet without significant nausea or vomiting [30]. GI-2 was determined by a retrospective review of daily entries by medical and nursing staff. Patients discharged prior to achieving GI-2 were considered to not have POI. Secondary outcomes included time to first stool, time to tolerance of oral diet, nasogastric tube (NGT) reinsertion and complications for both groups. Length of stay and 30-day complications (Clavien-Dindo grade, CD; Comprehensive complication index, CCI) were recorded [31, 32].

### Statistical methods

Statistical analysis was performed using SPSS 28.0 (SPSS Inc., Armonk, NY, USA). Numerical data are presented as median (IQR [range]) or mean (standard deviation) depending on parametricity identified with the Shapiro–Wilk test. Univariate analysis was performed using the Mann–Whitney *U* test for nonparametric variables or the Student *t* test for normally distributed continuous variables. The *χ*^2^ or Fisher’s exact test (when expected *n* < 5) was applied for categorical variables. All collected variables were used in the univariate logistic regression analysis. Statistically significant variables were then used for multivariate logistic regression analyses to determine predictors of POI. Data for multivariate logistic analyses were evaluated and met all assumptions. *P* values of less than 0.05 were considered statistically significant.

## Results

**Fig. 2:**
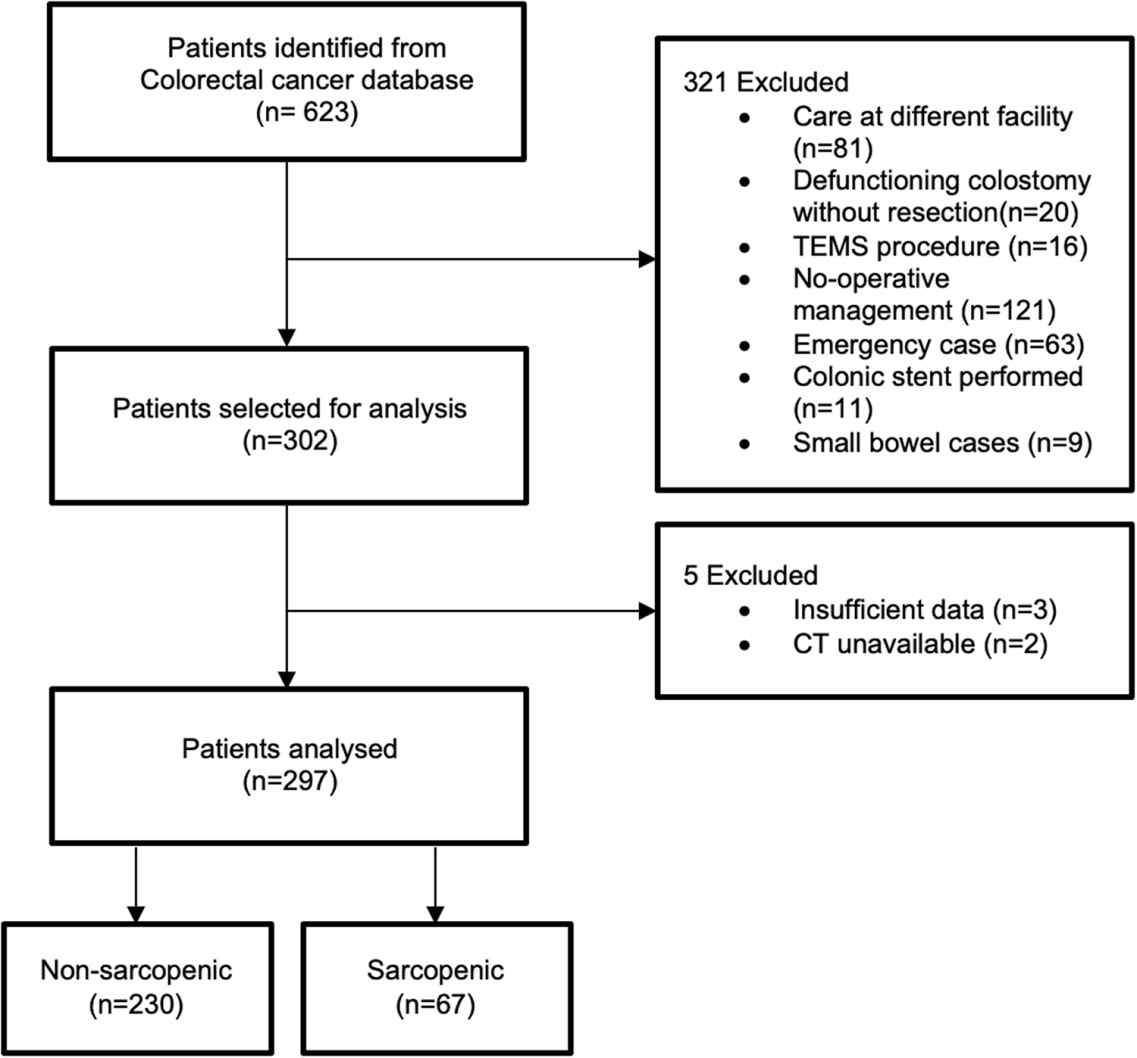
Patient selection flowchart

Of 297 patients included in the analysis, 22.6% (*n* = 67) were diagnosed with sarcopenia. Patient selection is reported in Fig. 2. Baseline characteristics demonstrate that the sarcopenic group was significantly older (74 vs. 68.5 years, *p* < 0.001) and showed no difference related to sex distribution (Table 1). The sarcopenic cohort has a significantly lower weight (72 vs. 80 kg, *p* < 0.001) and body mass index (24.4 vs. 28.8 kg/m^2^, *p* < 0.001). These patients were generally more comorbid with a greater proportion of ASA grade ≥ 3 (71.6% vs. 54.8%, *p* = 0.014) and a higher MFI-5 (44.8% vs. 31.7%, *p* = 0.048). No difference was found between rates of neoadjuvant therapy given between both groups (14.9% vs. 13.0%, *p* = 0.691). The sarcopenic group had lower preoperative hemoglobin, total protein and albumin levels (125 vs. 133.5, 71 vs. 73 and 35 vs. 37 g/L, respectively all *p* < 0.05). Table 2 demonstrates the perioperative features of the two groups. They shared similar surgical characteristics with respect to tumour site, AJCC stage, operative type or approach, and stoma formation or type. There were also no differences in intraoperative administration of opioid and intravenous fluid, or postoperative serum potassium.

**Table 1:** Comparison of baseline characteristics between patients with and without sarcopenia

	Non-sarcopenic (*n* = 230)	Sarcopenic (*n* = 67)	*p* value
Baseline characteristics			
Age, years	68.5 (58–76)	74 (67–82)	< 0.001
Gender			0.336
Female	101 (43.9)	25 (37.3)	
Male	129 (56.1)	42 (62.7)	
BMI, kg/m^2^	28.8 (24.9–31.9)	24.4 (22.2–28.6)	< 0.001
Weight, kg	80 (70–94)	72 (60–80)	< 0.001
Body composition			
TPA	2061.3 (1641.5–2684.1)	1092.8 (893.4–1430.6)	< 0.001
TPAI	720.5 (616.8–910.7)	382.70 (333.8–481.2)	< 0.001
ASA			0.014
I–II	104 (45.2)	19 (28.4)	
III–IV	126 (54.8)	48 (71.6)	
Modified Frailty Index-5			0.048
Low (0–1)	157 (68.3)	37 (55.2)	
High (≥ 2)	73 (31.7)	30 (44.8)	
Smoking history			0.987
Active	37 (16.1)	11 (16.4)	
Ex-smoker	78 (33.9)	22 (32.8)	
CCF	12 (5.2)	7 (10.4)	0.124
COPD	28 (12.2)	10 (14.9)	0.553
Hypertension	118 (51.3)	41 (61.2)	0.153
Diabetes mellitus			0.369
Prescribed tablets	47 (20.4)	17 (25.4)	
Prescribed insulin	6 (2.6)	0 (0.0)	
Prescribed regular steroids	6 (2.6)	2 (3.0)	> 0.999
Previous abdominal surgery	111 (48.3)	33 (49.3)	0.886
Neoadjuvant therapy given	30 (13.0)	10 (14.9)	0.691
Preoperative hemoglobin, g/L	133.5 (120–144)	125 (109–137)	< 0.001
Preoperative total protein, g/L	73 (69–77)	71 (67–75)	0.018
Preoperative albumin, g/L	37 (34–40)	35 (32–38)	< 0.001

Values are median (IQR) or number (proportion)

*ASA* American Society of Anesthesiologists physical status, *BMI* body mass index, *CCF* congestive cardiac failure, *COPD* chronic obstructive pulmonary disease

**Table 2:** Comparison of perioperative characteristics between patients with and without sarcopenia

	Non-sarcopenic (*n* = 230)	Sarcopenic (*n* = 67)	*P* value
Intraoperative characteristics			
Site of tumour			0.187
Colon	167 (72.6)	54 (80.6)	
Rectal	63 (27.4)	13 (19.4)	
AJCC stage*			0.979
0–2	141 (65.0)	43 (65.2)	
3–4	76 (35.0)	23 (34.8)	
Operations			0.224
Abdominoperineal resection	5 (2.2)	1 (1.5)	
Anterior resection	79 (34.3)	19 (28.4)	
Pan-proctocolectomy and total colectomy	24 (10.4)	6 (9.0)	
Hartmann’s procedure	14 (6.1)	0 (0.0)	
Left hemicolectomy	6 (2.6)	2 (3.0)	
Pelvic exenteration	10 (4.3)	4 (6.0)	
Right and extended right hemicolectomy	92 (40.0)	35 (52.2)	
Surgical approach			0.744
Open	77 (33.5)	21 (31.3)	
Laparoscopic	153 (66.5)	46 (68.7)	
Conversion from laparoscopic to open	31 (20.3)	7 (15.2)	0.445
Stoma formed post resection	62 (27.0)	16 (23.9)	0.615
Theatre duration, min	185.5 (143.8–230)	180 (126–240)	0.706
Postoperative characteristics			
Lowest postoperative serum potassium within POD 1–4, mmol/L	3.8 (3.6–4.2)	3.8 (3.5–4.1)	0.214
Intraoperative and recovery opioid use, MEQ	134 (90–179)	120 (95–166)	0.777
Total opioid use POD 1–4, MEQ	160 (77–334)	110 (40–290)	0.051
Total intraoperative fluids, ml	2000 (2000–2425)	2000 (1000–2000)	0.437
Total recovery fluids, ml	1000 (600–1300)	1000 (600–1225)	0.900

Values are median (IQR) or number (proportion)

*AJCC* American joint committee on cancer, *MEQ* morphine equivalents, *POD* postoperative day

*For adenocarcinoma

Postoperative outcomes are summarized in Table 3. POI rates (41.8% vs. 26.5%, *p* = 0.016) and NGT reinsertion rates were significantly higher in the sarcopenic group compared to the non-sarcopenic group (38.8% vs. 23.9%, *p* = 0.016). Median time to tolerance of oral diet (3 vs. 2 days, *p* = 0.047), first stool (4 vs. 3 days, *p* = 0.096), and GI-2 (4 vs. 3 days, *p* = 0.206) was prolonged by 1 day in the sarcopenic group. However, only tolerance to oral diet was found to be statistically significant. Overall complications were greater in the sarcopenic group. In addition to more frequent complications (85.1% vs. 68.3%, *p* = 0.007), patients with sarcopenia also had higher-grade complications (CD grade 3–5, 13.4% vs. 10.0%, *p* = 0.026 and CCI 22.6 vs. 12.2, *p* = 0.007). The sarcopenic cohort were more likely to have more than one complication; however, this did not reach significance (64.9% vs. 56.1%, *p* = 0.245). Following POI, patients with sarcopenia were more likely to have respiratory complications (20.9% vs. 11.3%, *p* = 0.043) and urinary tract infections (9.0% vs. 3.0%, *p* = 0.037). Anastomotic leak rate was higher in patients with sarcopenia although the difference was not statistically significant (7.5% vs. 2.6%, *p* = 0.064). Other complications such as intensive care unit admission were similar between the two cohorts. The 30-day mortality was low in both cohorts (1.5% vs. 0.8%, *p* = 0.537). A 1-day median increase in length of stay was seen in patients with sarcopenia (7 vs. 6 days, *p* = 0.013).

**Table 3:** Postoperative outcomes comparing patients with and without sarcopenia

	Non-sarcopenic (*n* = 230)	Sarcopenic (*n* = 67)	*P* value
Gastrointestinal recovery			
POI	61 (26.5)	28 (41.8)	0.016
NGT reinsertion	55 (23.9)	26 (38.8)	0.016
Time to tolerance of oral diet, days	2 (1–4)	3 (1–6)	0.047
Time to first stool, days	3 (2–4)	4 (2–5)	0.096
GI-2, days	3 (2–5)	4 (2–6)	0.206
Complications and clinical outcomes			
ICU admission	18 (7.8)	6 (9.0)	0.765
Complication	157 (68.3)	57 (85.1)	0.007
Multiple complications*	88 (56.1)	38 (64.9)	0.245
CCI	12.2 (0–22.6)	22.6 (8.7–29.6)	0.007
CD grade			0.026
No complication	73 (31.7)	10 (14.9)	
1–2	134 (58.3)	48 (71.6)	
3–5	23 (10.0)	9 (13.4)	
Respiratory complication	26 (11.3)	14 (20.9)	0.043
Urinary tract infection	7 (3.0)	7 (9.0)	0.037
Acute kidney injury	16 (7.0)	8 (11.9)	0.188
Cardiac complication	14 (6.1)	3 (4.5)	0.771
DVT/VTE	1 (0.4)	1 (1.5)	0.401
Electrolyte disturbance	53 (23.0)	21 (31.3)	0.167
Anastomotic leak	6 (2.6)	5 (7.5)	0.064
Blood transfusion required	14 (6.1)	4 (6.0)	0.972
Return to theatre within 30 days	8 (3.5)	5 (7.5)	0.161
Readmission within 30 days	13 (5.7)	6 (9.0)	0.331
Mortality within 30 days	2 (0.8)	1 (1.5)	0.537
Length of stay, days	6 (4–8)	7 (5–12)	0.013

Values are median (IQR) or number (proportion)

*CCI* comprehensive complication index, *CD* Clavien-Dindo grade, *DVT* deep vein thrombosis. *ICU* intensive care unit, *NGT* nasogastric tube, *POI* postoperative ileus, *VTE* venous thromboembolism

*Only for patients who had a complication

Following univariate analysis, shown in Table 4, several factors were predictive of POI including sarcopenia, male sex, ASA grade ≥ 3, smoking history, previous abdominal surgery, postoperative hypokalemia, open surgical approach, postoperative day 1–4 opioid use greater than median, intensive care unit admission and CD grade ≥ 3. However, on multivariate analysis, sarcopenia (OR 2.0, 95% CI 1.1–3.8, *p* = 0.029), male sex (OR 1.9, 95% CI 1.0–3.5, *p* = 0.036), postoperative hypokalemia (OR 3.2, 95% CI 1.6–6.5, *p* = 0.001) and postoperative opioid use remained strongly predictive of POI (OR 2.4, 95% CI 1.3–4.3, *p* = 0.004).

**Table 4:** Univariate and multivariate logistic regression analysis for values predictive of postoperative ileus

	Odds ratio	95% CI	*P* value
Univariate logistic regression			
Sarcopenia	1.989	1.128–3.506	0.017
Males	1.808	1.075–3.042	0.026
ASA > 3	1.832	1.085–3.093	0.024
Smoking history	1.871	1.129–3.102	0.015
Previous abdominal surgery	1.893	1.143–3.134	0.013
Postoperative hypokalemia (< 3.5 mmol/L)	3.164	1.695–5.907	< 0.001
Open approach	2.263	1.350–3.792	0.002
Postoperative opioid use > median	2.826	1.690–4.725	< 0.001
Intensive care unit admission	3.063	1.315–7.135	0.009
CD complications > 3	3.513	1.660–7.433	0.001
Multivariate logistic regression			
Sarcopenia	2.020	1.073–3.802	0.029
Male	1.909	1.043–3.495	0.036
ASA ≥ 3	1.481	0.827–2.652	0.187
Smoking history	1.460	0.825–2.582	0.194
Previous abdominal surgery	1.676	0.945–2.971	0.077
Postoperative hypokalemia (< 3.5 mmol/L)	3.229	1.603–6.506	0.001
Open approach	2.376	0.994–3.231	0.052
Postoperative opioid use > median	2.376	1.328–4.250	0.004
Intensive care unit admission	0.490	0.084–2.864	0.428
CD complications ≥ 3	3.142	0.662–14.915	0.150

Variables not reaching significance: age ≥ 65, BMI ≥ 30, MFI-5 ≥ 2, CCF, COPD, HTN, DM, steroid use, preoperative anemia and hypoalbuminemia, stoma formation, laparoscopic converted to open, theatre duration > 3 h, rectal cancer, blood transfusion, return to theatre

*ASA* American Society of Anesthesiologists physical status, *BMI* body mass index, *CCF* congestive cardiac failure, *CD* Clavien-Dindo grade, *COPD* chronic obstructive pulmonary disease, *DM* diabetes mellitus, *HTN* hypertension requiring medication, *MFI* modified frailty index

## Discussion

Our results confirm that in our cohort of colorectal surgical patient’s sarcopenia is associated with increased morbidity, consistent with previously reported data in the literature. However, our study demonstrates a strong association between preoperative radiologically diagnosed sarcopenia and POI by validated criteria following colorectal surgery. Of our patients with newly diagnosed colorectal cancer, 22.6% were found to have sarcopenia. This is similar to our previously reported cohort of patients with locally advanced rectal cancer [3, 33]. Although our rate is lower than published pooled results of 37% found in a recent meta-analysis, this may be explained by other studies using various methods to establish sarcopenia, the inclusion of emergent cases, and variable international ethnicities and demographics.

Overall, we found 41.8% of patients with sarcopenia developed POI. Defining POI is contentious, with no one accepted method. Defining POI using GI-2 has a reported rate of 10.1–34.5%, shown in the literature and in our previous studies [17, 34, 35]. Owing to the variable definitions, earlier studies have not looked at the relationship between POI and sarcopenia in detail. Three papers found a low incidence of postoperative ileus in patients with and without sarcopenia (< 6%) [21, 22, 24], with only one paper demonstrating a higher incidence associated with patients with sarcopenia [23]. The paper closest to our own study, that by Sasaki et al., demonstrated an overall rate of POI of 9.9%, with 19.1% in the sarcopenic cohort developing POI [25]. In our study, the strict definition using a validated composite measure demonstrated a clear correlation between POI and sarcopenia. Our study further corroborates this by the higher requirement for reinsertion of NGT seen in the sarcopenic cohort.

One other colorectal-specific study has investigated the predictive value of a low SMI for POI [25]. Sasaki et al. calculated SMI bioelectrical impedance analysis preoperatively for 213 patients and diagnosed POI using a clinical definition of POI, albeit not a validated measure. Despite using an alternative method of diagnosing low skeletal muscle mass, they demonstrated a difference in the prevalence of POI between low SMI and normal SMI (19.1% vs. 5.5%, *p* = 0.005). On multivariate analyses for 78 matched patients, SMI remained a predictor of POI (OR 10.80, 95% CI 1.25–93.20, *p* = 0.031). Our findings along with the results of Sasaki et al. suggest that there is an association between sarcopenia and POI, and that both SMI and TPAI have proven useful prognostic markers for clinicians to identify those at risk of POI.

The association between sarcopenia and POI may be multifactorial. As speculated by Rinaldi et al. [20], patients with sarcopenia have a nutritional imbalance that predisposes them toward POI. Previous studies, including our present study, found sarcopenia is associated with signs of nutritional deficiencies such as decreased hemoglobin levels, serum total protein and albumin [36]. Nutritional imbalance may lead to a pro-inflammatory state, impairing macrophage response to irritation of the peritoneum [20]. This pro-inflammatory state is suspected to contribute to increased complications for patients with sarcopenia [37]. Additionally, smooth muscle contractility is impaired in patients with colorectal cancer as a result of the accumulation of collagen around the intestinal plexus [25]. Furthermore, POI and sarcopenia, separately, have been correlated to elderly and comorbid patients [18, 38, 39]. In our study, despite age and comorbidities being higher in the baseline characteristics for the sarcopenic group, age was not predictive of POI in univariate analyses. ASA grade ≥ 3, only reached significance on the univariate analysis. With sarcopenia being independently predictive of POI, we suggest that an association exists. Besides sarcopenia, male sex, increased opioid use postoperatively, and hypokalemia were also predictive of POI, which is consistent with previous literature [9, 28, 39].

Sarcopenia results in poor short- and long-term postoperative outcomes. In a recent systematic review, patients with colorectal cancer and sarcopenia had an increase in cardiopulmonary complications (OR 2.92, 95% CI 1.96–4.37), severe postoperative complications (OR 1.72, 95% CI 1.10–2.68), and mortality (OR 3.21 95% CI 2.01–5.11) [3]. We also demonstrate that patients with colorectal cancer and sarcopenia had a significant increase in CCI, higher-grade CD complications and respiratory complications. We also found a significant increase in patient’s length of stay. However, this only represented a median increase of 1 day. Interestingly, despite a higher incidence of anastomotic leaks in the sarcopenic group, this did not reach significance. This result is consistent with the pooled results of the meta-analysis; however, in our study, as a result of our sample size and the low overall incidence of anastomotic leaks (3.7%) a type II error cannot be ruled out [3].

Identifying frail patients helps clinicians provide accurate information about the risk of complications to patients as well as guide interventions, such as nutritional support and physiotherapy. Highlighted in this study is TPAI as an easy method to assess colorectal surgical patients. The method of lean muscle mass assessment described by Jones et al. has been shown to be a satisfactory surrogate marker for sarcopenia [3, 8] and allows an easy and convenient measure of lean muscle mass assessment in a cohort of patients that predictably have staging scans available [4, 8]. Assessment of total psoas area represents an easy opportunity to identify patients at risk of postoperative complications, helping clinicians to provide accurate information about surgical risk to their patients. Additionally identifying at-risk patients can prompt clinicians to take early action to treat POI and avoid secondary complications such as respiratory complications or aspiration pneumonia, which was notably higher in the POI group.

Given the results from Sasaki et al. and our findings, methods of modifying sarcopenia and the impact on POI rates could be further explored. However, there are limited reports of prehabilitation programmes that have successfully modified sarcopenia and resulted in improved rates of postoperative complications [40]. To our knowledge, no studies have looked to improve POI rates with prehabilitation, particularly during patients’ neoadjuvant therapy. In a study of colorectal surgical patients undergoing neoadjuvant therapy, a 13- to 17-week guided walking programme improved lean muscle mass [20]. In our study, albeit a small portion of our cohort, 10 of the 13 rectal cancers that were sarcopenic had undergone neoadjuvant therapy. With neoadjuvant therapy now favoured in treating rectal cancers [41], this represents a potential opportunity for prehabilitation to improve postoperative outcomes, like POI, for these patients.

Although demonstrating an association between sarcopenia and POI, this study had several limitations. This study was retrospective and single centred in design. As a result of the limitation of demographic recording, patients in our hospital may declare if they are Aboriginal-Torres Strait Islanders (ATSI) or Non-ATSI. We could not delineate ethnicity further, and the proportion of ATSI patients was too small to make a meaningful analysis. TPAI is used as a marker of sarcopenia, and does not account for myosteatosis, as well as physical tests for sarcopenia. As a result of the paucity of data available, this study was designed as an observational study without a dedicated sample size. Although this study suggests a link between sarcopenia and POI, a prospective multicentred study incorporating physical tests for sarcopenia is required to confirm this positive association.

## Conclusion

Sarcopenia demonstrates a significant association with POI and postoperative complications. Future research towards identifying sarcopenia’s impact on complications would improve clinicians' ability to more accurately consent patients for the risk of surgery and assist in operative planning.

## Data Availability

Not applicable.
